# Capsule production promotes Group B *Streptococcus* intestinal colonization

**DOI:** 10.1128/spectrum.02349-23

**Published:** 2023-09-21

**Authors:** Michelle J. Vaz, Sophia Dongas, Adam J. Ratner

**Affiliations:** 1 Department of Pediatrics, NYU School of Medicine, New York, New York, USA; 2 Department of Microbiology, NYU School of Medicine, New York, New York, USA; Vanderbilt University Medical Center, Nashville, Tennessee, USA

**Keywords:** *Streptococcus agalactiae *(Group B *Streptococcus)*, capsule serotype, murine model

## Abstract

Late-onset disease is the most common clinical presentation of Group B *Streptococcus* (GBS) infection during infancy, and gastrointestinal (GI) colonization is an important precursor. Previously, we described a murine model of postnatal GBS GI colonization that resulted in sustained colonization and progression to invasive disease. Capsular polysaccharide is an important GBS virulence factor. Vaccines based on a subset of capsular serotypes are in clinical trials. However, little is known regarding the role of specific GBS capsular serotypes in GI colonization. We examined the role of GBS capsule in GI colonization using capsule-producing and acapsular strains derived from GBS strain A909 (serotype Ia) in a murine model. Using isogenic GBS strains differing only in capsular serotypes, we explored the role of specific serotypes in GI colonization by determining competitive indices during cocolonization. We found that GBS A909 colonizes the murine GI tract without causing invasive disease. In monocolonization experiments, there was colonization persistence with the capsule-producing strain (100%) compared to the acapsular mutant strain (13%). In cocolonization experiments, the capsule-producing strain outcompeted its isogenic acapsular mutant, with a geometric mean competitive index of 8, 95% confidence interval (CI) [1.7, 38.9] in the colon at 7 days post-colonization. A909 expressing its native serotype Ia capsule outcompeted an isogenic mutant that expresses serotype III capsule, with a geometric mean competitive index of 2.5, 95% CI [1.2, 5.1] in the colon at 7 days post-colonization. Thus, polysaccharide capsule production enhances GBS GI colonization *in vivo*. In an A909 genetic background, the production of a serotype Ia capsule provides a competitive advantage over an isogenic strain producing type III capsule. The murine model is a valuable tool to understand the role of GBS capsule types in GI colonization.

**IMPORTANCE:**

The establishment of GBS intestinal colonization is believed to be a critical precursor to late-onset disease in neonates, which has a significant impact on neurodevelopment outcomes in this population. Our prior work described a murine model of postnatal Group B *Streptococcus* (GBS) acquisition and invasive disease. Using this model, we explored the importance of GBS polysaccharide capsule production on gastrointestinal colonization. We found that the expression of capsule (compared to isogenic acapsular strains) provides an advantage in intestinal colonization and, importantly, that capsule type Ia has an advantage over capsule type III in a GBS A909 strain background. We speculate that specific serotypes may differ in colonization fitness, which may play a role in serotype distribution in neonatal disease.

## INTRODUCTION

*Streptococcus agalactiae* [Group B *Streptococcus* (GBS)] is a leading cause of neonatal sepsis (1). Widespread implementation of intrapartum antibiotic prophylaxis (IAP) of GBS colonized mothers has reduced the incidence of early-onset infection (occurring in the first week of life) (2). However, rates of maternal colonization and incidence of late-onset (LO) infection (≥7 days of life) remain unchanged (3). LO infection is now the most common manifestation of invasive GBS disease in infants (4), with an overall case fatality rate of 2% to 7%, which is notably higher (22%) in low-birthweight infants (5). The sequelae of LO infection include meningitis (31–59%), long-term neurodevelopmental sequelae in up to 50%, and moderate to severe neurodevelopmental impairment in approximately 23% of survivors (6 – 10).

Maternal rectovaginal colonization is an important risk factor for LO disease. Approximately 50% of infants who go on to develop LO disease are colonized at birth with the same GBS serotype as their mother (11). Recent reports evaluating GBS colonization in mother-baby dyads showed colonization in 23% of infants at 2 months, with 95% concordance between maternal and infant GBS serotypes (12). Among infants with LO disease, gastrointestinal (GI) colonization appears to be a critical precursor. Studies looking at longitudinal stool cultures of preterm infants showed the predominance of bacteria in the stool just prior to bacteremia and invasive disease, suggesting that the organism invades the host from the gut in late-onset disease (13, 14).

Despite its importance, there are no effective strategies to reduce the incidence of LO disease or to identify those infants who may be most at risk. Vaccination strategies are being explored to reduce the GBS disease burden. GBS virulence factors that aid in colonization, invasion, and immune evasion are being used as potential vaccine candidates in various stages of preclinical and clinical trials. Maternal immunization with protein-conjugated GBS capsular polysaccharides may reduce disease risk in neonates and young infants in a serotype-specific manner (15 – 18). However, the role of capsule in GI colonization has not been explored.

Our prior work described a murine model of postnatal GBS acquisition, that resulted in sustained GI colonization and, in sporadic cases, progression to invasive disease, recapitulating GBS acquisition in neonates (19). Using this model, we used isogenic GBS capsular and acapsular strains to investigate the role of capsule on the kinetics of intestinal colonization. We also used isogenic GBS strains differing only in capsule type to determine the role of specific capsule types using competition indices and immunohistochemistry.

## MATERIALS AND METHODS

All experiments were performed in accordance with protocols approved by the NYU School of Medicine’s Institutional Animal Care and Use Committee (IACUC).

### Bacterial strains and growth conditions

GBS wild-type (WT) strains A909 (serotype Ia), its derivatives, and COH1 were grown to stationary phase at 37°C in trypticase soy (TS) broth and enumerated by plating on TS agar or CHROMagar StrepB plates. Previously described capsule-deficient (A909Δ*cpsE),* capsule switch (CS) (A909 strain expressing type III capsule), and revertant strains (Rev) (A909 strain expressing native Ia capsule) were used (20).

### GI colonization model

Adult male and female C57BL/6J mice (8–12 weeks) were purchased from Jackson Laboratories (Bar Harbor, Maine), given at least 3 days to acclimate to the local facility, and mated as pairs and trios. Dams were monitored for the birth of litters, and animals aged 12–14 days were designated as preweaning mice in this cohort.

For the monocolonization model with WT A909 (Ia) and capsule-deficient (A909Δ*cpsE*) strains, bacterial cultures were grown overnight to the stationary phase. Following overnight growth, the optical densities of the two strains were normalized. Cultures were then centrifuged and resuspended in sterile Dulbecco’s phosphate-buffered saline (DPBS) to a final concentration of 10^9^ CFU/mL. Animals were orally fed using a sterile feeding tube with 10^8^ CFU of GBS resuspended in 50 µL of DPBS, respectively, and remained with their biologic, non-colonized dam. The animals were monitored daily for signs of illness and mortality. Rectal swabs were collected at predetermined time intervals (7, 14, 21, and 28 days post-oral inoculation), and serial dilutions were plated for determination of GBS CFUs on chromogenic agar.

For the cocolonization model, bacterial cultures were grown and centrifuged as described above. Cultures were resuspended in PBS, and a 1:1 mixture was made of the two resuspended strains to be competed. Mice were orally infected as described in monocolonization experiments, housed with their biological dams, and monitored daily for signs of illness and mortality. At predetermined time points (3, 7, and 14 days post-infection), animals were euthanized, and the GI tract was harvested. Each portion (small intestine, cecum, and colon) was homogenized using a Next Advance Bullet Blender Storm 24 Place Bead Homogenizer. Serial dilutions were plated on CHROMagar StrepB for the determination of GBS CFUs. In addition to plating serial dilutions, dilutions were spread on CHROMagar plates with glass beads for the determination of serotype by colony immunoblot. In additional cohorts, preweaning mice were similarly inoculated, and rectal swabs were obtained longitudinally.

### Colony immunoblotting

Following growth at 37°C, colonies were counted to identify plates with 20–200 colonies for subsequent immunoblotting. Plates were briefly overlaid with nitrocellulose membranes (Amersham) to allow adherence of GBS material to the membrane. Membranes were blocked in 3% bovine serum albumin (BSA) in PBS (blocking solution) for 1 hour. Blots were then transferred to type Ia or type III *Streptococcus* group B type antisera (Statens Serum Institut) and incubated for 30 minutes with gentle shaking. Type Ia antiserum was diluted 1:2,000 in the blocking solution, and type III antiserum was diluted 1:5,000 in the blocking solution. Blots were then washed three times in PBS. Blots were incubated in horseradish peroxidase (HRP)-conjugated goat anti-rabbit secondary antibody (Pierce) diluted 1:1,000 in a blocking solution for 2 hours. Blots were washed three times in PBS and stained using a 3,3′-diaminobenzidine tetrahydrochloride substrate kit (Abcam). Colonies positive for each capsule type were counted. In experiments comparing WT to acapsular mutants, nonreacting GBS colonies representing the acapsular mutants were also counted. Competitive index (CFU strain 1 recovered/CFU strain 1 inoculated)/(CFU strain 2 recovered/CFU strain 2 inoculated) was calculated and log-transformed for calculation of geometric mean and 95% confidence interval (CI) for each condition and time point.

### Microscopy and staining

Preweaning animals were euthanized at predetermined time points, and the distal small intestine (1 cm segment) and distal colon were removed and fixed in 4% paraformaldehyde. The NYU Langone’s Experimental Pathology/Immunohistochemistry Core Laboratory embedded all fixed tissues in paraffin and then serially sectioned onto slides. NYU Experimental Pathology Immunohistochemistry Core Laboratory performed hematoxylin and eosin (H&E) staining using the Leica ST5020 Multistainer in conjunction with the Leica CV5030 Coverslipper for optimized workflow. Slides were scanned at 40× resolution using the Leica SCN400 whole slide scanner. Immunofluorescent labeling of GBS was performed following deparaffinization and rehydration of sectioned tissue. Heat-induced epitope retrieval was performed as per the manufacturer’s recommendations (Abcam). Nonspecific binding sites were blocked with 10% normal goat serum and 1% bovine serum albumin (Sigma). Rabbit anti-GBS polyclonal antibody (Abcam ab53584, 1:200 dilution) was applied. Following serial washes with PBS + 0.025% triton X-100, Alexa Fluor 647 goat anti-rabbit immunoglobulin G (IgG; Invitrogen; 1:500 dilution) was added for 30 minutes in the dark. Slides were counterstained using Hoechst 33342 (Invitrogen). Coverslips were mounted with Vectashield Hardset mounting medium (Vector Laboratories), and slides were stored at 4°C. Slides to which no primary antibody or no secondary antibody was added served as negative controls. Slides were scanned at 20× resolution using the Hamamatsu NanoZoomer 2.0HT scanner.

### Statistical analysis

Statistics were calculated using GraphPad Prism 9 Software. The persistence of GI tract monocolonization between WT A909 (Ia) and A909 *cpsE* KO was compared using the Log-rank (Mantel-Cox) test.

## RESULTS

### Capsule is important for GI colonization

**Fig 1:**
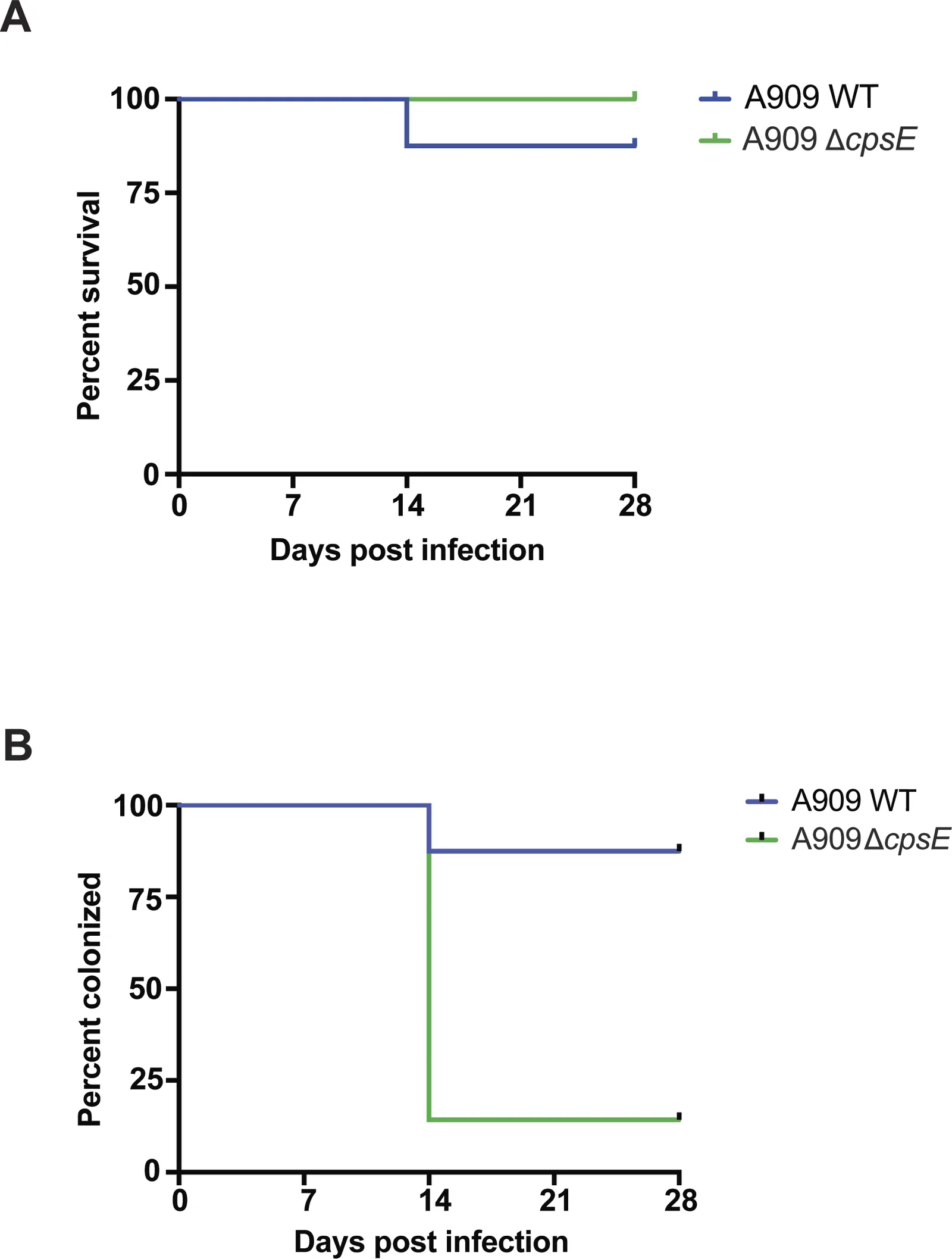
Establishing the role of capsule in GI colonization. (A) Kaplan-Meier survival curve of preweaning C57BL/6J mice orally infected with A909 WT (*n* = 8) and A909Δ*cpsE* (*n* = 7) in separate cohorts *(P = NS, log-rank)*. (B) Colonization duration as determined by longitudinal examination of rectal sampling in preweaning mice (*n* = 8 in A909 WT cohort, *n* = 7 in A909Δ*cpsE*, two representative litters) (*P ≤ 0.006, log-rank*). The reported data are derived from two independent experiments.

We orally infected preweaning mice (12–14 days) with A909WT (capsule producing) and A909Δ*cpsE* (capsule deficient) in separate cohorts. Animals were monitored daily for signs of illness. Rectal swabs were performed at 7-day intervals to assess GI colonization. Animals infected with A909 WT had a survival of 88% compared to 100% in the A909Δ*cpsE*-infected cohort (*P = ns, log-rank*) (Fig. 1A). On assessing the colonization data, we noted persistent colonization in 100% of survivors in the WT strain cohort at 28 days post-infection, whereas only 13% of animals infected with the KO strain remained colonized at 14 days post-infection (*P =< 0.006, log-rank*) (Fig. 1B).

### Capsule-producing strains outcompete acapsular strains in a cocolonization model

**Fig 2:**
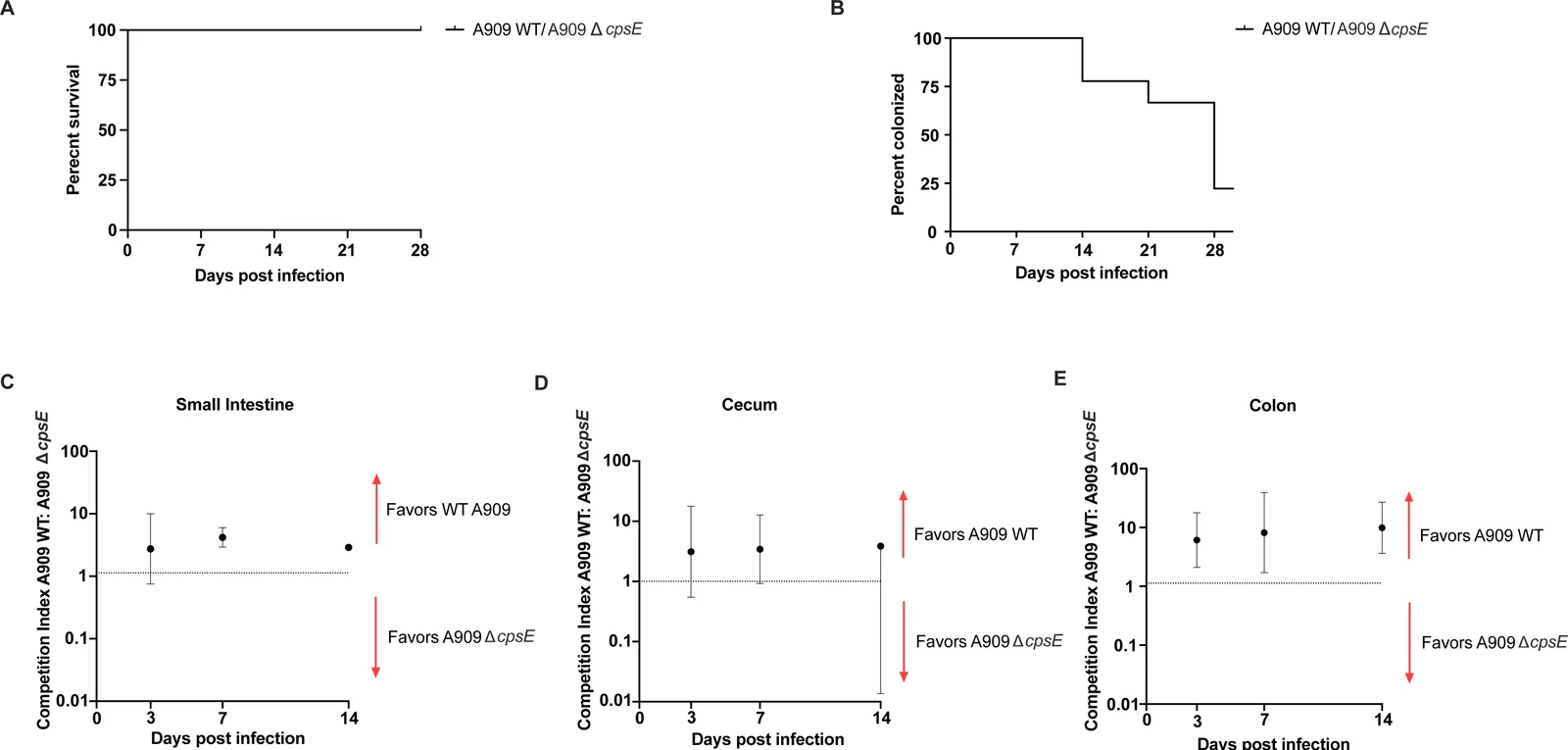
Capsule-producing A909 outcompetes capsule-deficient A909 in a murine model of GI cocolonization. Preweaning C57BL/6J mice were orally inoculated with a 1:1 mixture of A909 WT and A909Δ*cpsE* in two separate experiments. (**A, B)** In the first experiment (*n* = 8), animals were followed longitudinally to assess survival. The Kaplan-Meier curve shows 100% survival. In addition, Kaplan-Meier curve demonstrates colonization persistence up to 28 days post-infection as determined by longitudinal rectal sampling. (**C–E)** In the second experiment, the GI tract, small intestine, cecum, and colon were harvested at predetermined time intervals. Immunoblot with type Ia primary antibody was used to differentiate between capsule A909 WT and A909 Δ*cpsE*. Data points represent geometric mean competition indices and error bars represent 95% confidence intervals.

We assessed the fitness of encapsulated A909 WT (serotype Ia) vs A909Δ*cpsE* in a GI cocolonization model. Preweaning mice (12–14 days) were orally infected as described in separate experimental groups. In the first group, animals were monitored for survival and colonization status. There was 100% survival in this experimental cohort, with some animals (22%) remaining colonized until 28 days post-infection as determined by rectal sampling (Fig. 2A and B). In the second experimental group, animals were similarly infected and were monitored daily for signs of illness. They were euthanized at 3, 7, and 14 days post-infection, and the GI tract was harvested. In the small intestine, the capsule-producing strain outcompeted its isogenic capsule-deficient mutant at certain time points with a geometric mean competitive index of 4, 95% CI [2.9, 5.9] at 7 days post-colonization (Fig. 2C). In the cecum, the geometric mean point estimate was greater than 1 at all time points; however, the CI crossed one. The highest geometric mean competitive index in the cecum was 3.4, 95% CI [0.9, 12.7] at 7 days post-colonization (Fig. 2D). In the colon, the capsule-producing strain outcompeted its isogenic capsule-deficient mutant at all time points, with geometric means of 6, 95% CI [2.1, 17.8]; 8.2, 95% CI [1.7, 39]; and 9.9, 95% CI [3.6, 27.8] at 3, 7, and 14 days post-colonization, respectively (Fig. 2E). This dominance was also noted on rectal swabs obtained in the first cohort (Fig. S1). This suggests a sustained competitive advantage of capsule-producing A909 over the capsule-deficient A909Δ*cpsE* throughout the GI tract. Microscopic examination of the colon in the cocolonization cohort demonstrated GBS via immunofluorescence. A robust signal was detected in the lumen, likely attributed to abundant GBS within fecal material; however, a sparse GBS signal was also noted within the submucosa. H&E staining showed normal appearing epithelial architecture (Fig. 4A and B)**.**

### Serotype Ia capsule provides a competitive advantage over an isogenic strain producing a type III capsule

**Fig 3:**
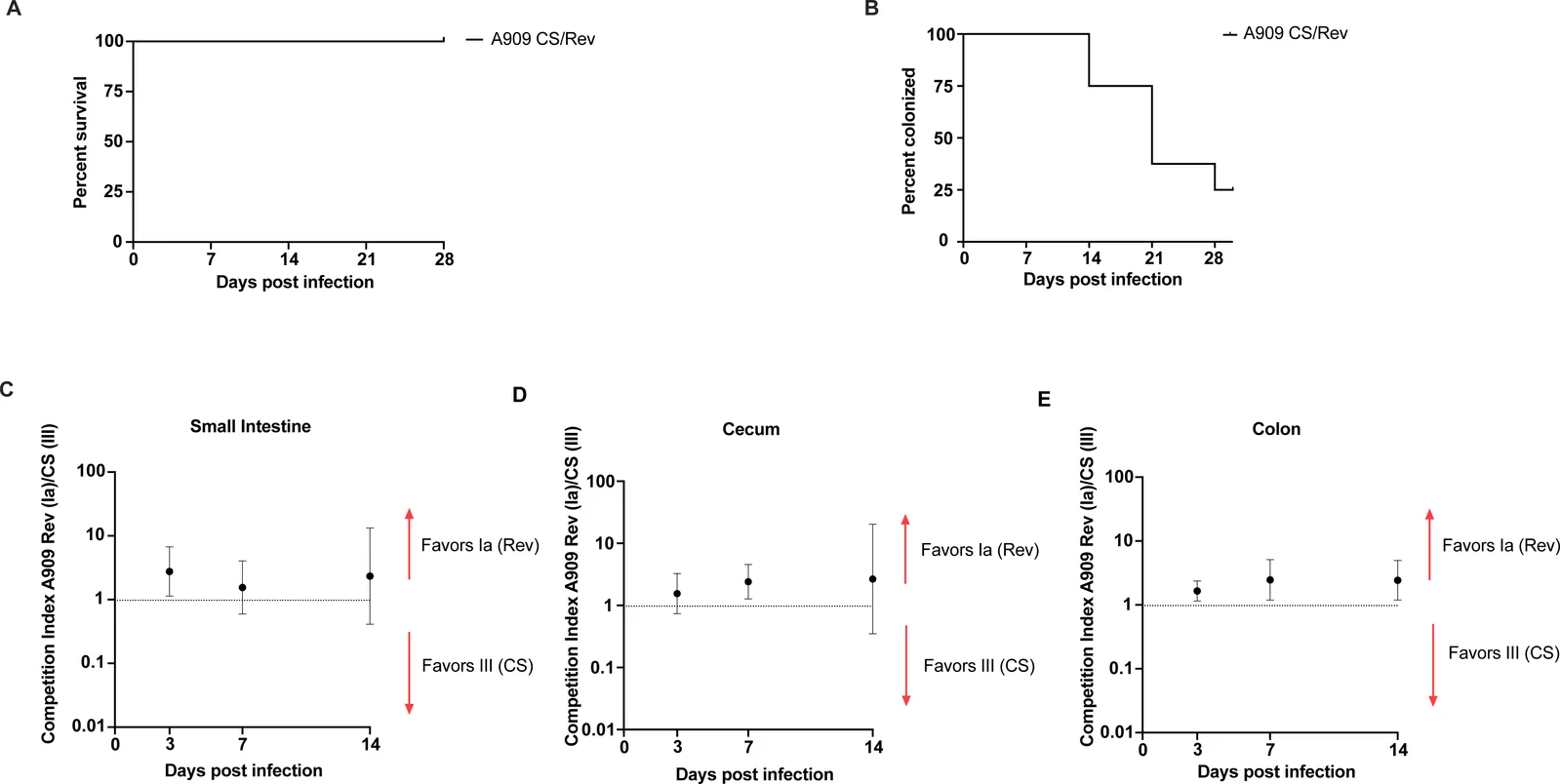
GBS A909 expressing its native serotype Ia capsule outcompetes an isogenic mutant that expresses a serotype III capsule. Preweaning C57BL/6J mice were orally inoculated with a 1:1 mixture of A909 Rev and A909 CS in two separate experiments. (**A, B)** In the first experiment (*n* = 9), animals were followed longitudinally to assess survival. The Kaplan-Meier curve shows 100% survival. In addition, Kaplan-Meier curve demonstrates colonization persistence up to 28 days post-infection as determined by longitudinal rectal sampling. (**C–E) **In the second experiment, the GI tract, small intestine, cecum, and colon were harvested at predetermined time intervals. Immunoblot with type Ia and type III primary antibody was used to differentiate between capsule A909 Rev and A909 CS. Data points represent geometric mean competition indices and error bars represent 95% confidence intervals

**Fig 4:**
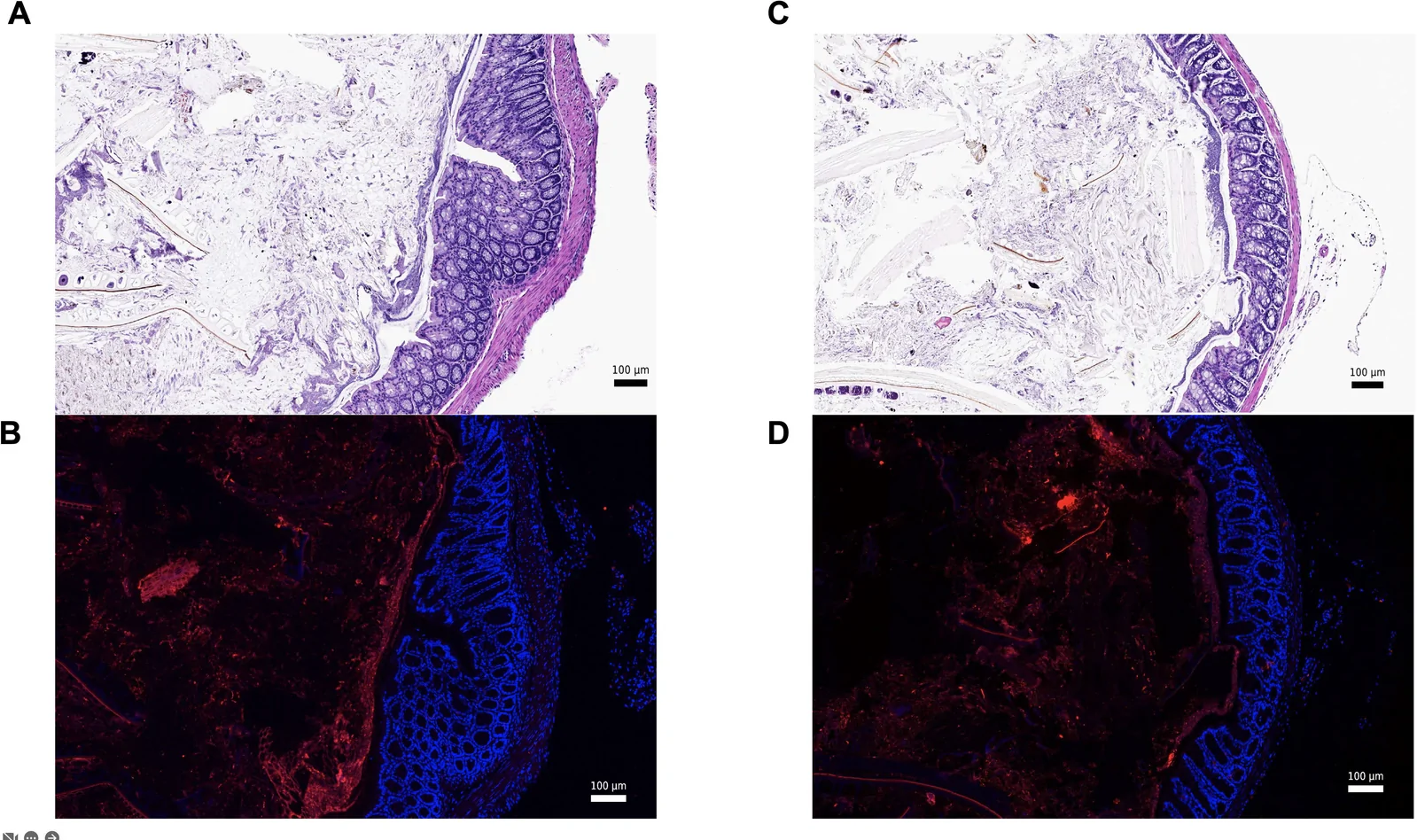
GBS A909 colonizes the GI tract. (A, B) Representative images of H&E and GBS-specific immunofluorescent antibody staining of the colon in mice cocolonized with A909 WT and A909Δ*cpsE* at day 14 post-infection, respectively.** (C, D)** Representative images of H&E and GBS-specific immunofluorescent antibody staining of the colon in mice cocolonized with A909 ReV and A909 CS at day 14 post-infection, respectively. GBS are visualized using a GBS-specific antibody stain (red) and cell nuclei are stained with DAPI (blue).

We competed matched capsule switch (CS) (A909 strain expressing type III capsule) and revertant (Rev) (A909 strain expressing native type Ia capsule) strains in the murine GI cocolonization model to further probe the role of these two capsular serotypes. We previously confirmed that these strains produce similar amounts of capsule (20). Preweaning animals (12–14 days) were orally infected with a 1:1 mixture of both strains in two separate experimental groups. In the first cohort, animals were monitored for survival and colonization status. There was 100% survival in this experimental cohort, with some animals (25%) remaining colonized until 28 days post-infection (Fig. 3A and B). In the second experimental group, the GI tract was harvested as described above to assess competition indices. In the small intestine, the A909 Rev strain expressing native Ia capsule outcompeted the capsule switch strain at day 3 post-colonization with a geometric mean of 2.8, 95% CI [1.1, 6.7] at 3 days post-colonization. At days 7 and 14 post-colonization, we also noted a geometric mean > 1, the CI crossed 1 (Fig. 3C). Similarly, in the cecum, the competition index point estimate was greater than 1 at all time points with the CI crossing 1 at days 3 and 14 post-infection. The highest geometric mean in the cecum was 2.4, 95% CI [1.3, 4.6] at 7 days post-colonization (Fig. 3D). In the colon, the A909 Rev strain expressing native Ia capsule outcompeted the capsule switch at all time points, with geometric means of 1.7, 95% CI [1.1, 2.4]; 2.5, 95% CI [1.2, 5.1]; and 2.4, 95% CI [1.2, 5] at 3, 7, and 14 days post-colonization, respectively (Fig. 3E). This advantage was also noted on rectal swabs performed in the first cohort in this experimental group (Fig. S2A). These findings suggest a competitive advantage of capsule-producing serotype Ia over capsule-producing serotype III in the GI tract. Taken together, these data suggest that in the same genetic background, the production of type Ia capsule may confer an advantage over type III capsule in GI tract colonization. To determine whether this advantage persisted with strains in their natural genetic background, preweaning mice were orally infected with a 1:1 mixture of A909 WT and COH1 (serotype III) in a separate cohort. Rectal swabs were performed at 7-day intervals for immunoblotting. Under these circumstances, serotype Ia confers an advantage over serotype III ( Fig. S2B). On immunofluorescence examination of the colon in the cocolonization cohort, we demonstrate GBS localization within the lumen, likely due to a predominance in fecal matter, and sometimes within the epithelial and submucosal layers. H&E staining again demonstrated normal appearing epithelial architecture in the colon (Fig. 4C and D). No fluorescent signal was detected in biological and technical controls (Fig. S3A and B).

## DISCUSSION

GBS intestinal colonization is important for the pathogenesis of LO disease in infants (11, 21). Weindling et al. showed that 40% of infants who were colonized at birth continued to have intestinal carriage at 12 weeks of life (21). A study of GBS colonization dynamics in women during and after pregnancy and in their infants noted that on rectal sampling at 1 year of age, infants were more often GBS carriers and colonized by the same clone if their mothers were carriers (22). Recent work in a cohort of preterm infants in the NICU also showed gut pathogen colonization preceded bacteremia (14). Metagenomic sequencing on stool sample demonstrated that 15 days prior to bacteremia, the gut microbiome harbored the future bacteremic species in the most abundant quartile in ~50% of the cases. This shows that pathogens invade the host from the gut, and GI colonization is a critical precursor to LO disease.

Previously described murine models of GBS GI colonization have evaluated the role of critical bacterial factors, including SrtA sortase and HvgA adhesin, needed for adhesion to and invasion of the GI epithelial surfaces (23, 24). The neonatal intestine and its microbiota exhibit enhanced permissiveness to GBS, easing GBS gut colonization, and translocation across the gut-vascular barrier, with subsequent bacteremia and meningitis (25). Additional studies have successfully modeled the GI colonization phenotype with the development of invasive disease after oral inoculation of GBS and our prior work described a model of LO GBS disease after oral inoculation with prolonged gastrointestinal colonization and development of invasive disease in 27% of cases (19, 26).

GBS produces capsule, which is an important virulence factor. The capsule is represented by 10 different serotypes (Ia, Ib, II-IX). The most frequent GBS serotype-causing disease in infants is serotype III (61.5%), followed by serotypes Ia (19.1%), V (6.7%), and Ib (5.7%) (27). The importance of capsule in GBS disease pathogenesis has been well established in *in vivo* and *ex vivo* work. The polysaccharide capsule enhances GBS virulence by facilitating evasion of the innate immune response by different mechanisms (28, 29). Noble et al. showed that the presence of a GBS capsule aids in GBS colonization and invasion of the gravid reproductive tract (30). Expression of type Ia or type III capsule confers a fitness advantage in GBS vaginal colonization over acapsular strains of the same genetic background (20). Here we showed that capsule is important for the establishment and maintenance of GBS GI colonization. Consistent with prior findings in a vaginal colonization model, we also demonstrated that type Ia capsule confers an advantage over type III capsule in an A909 background in cocolonization. This observation was surprising given that type III capsule is clinically more prevalent in invasive disease, suggesting that non-capsule elements like adhesins (Srr-2, HvgA) may contribute to colonization outcomes.

Finally, the impact of GBS vaccines that target a subset of capsule types on GI colonization has not been explored. Capsule switching and serotype replacement have been described in GBS, which may lead to an increased prevalence of non-vaccine serotypes (NVTs), a phenomenon described in in *Streptococcus pneumoniae* (31 – 34). Therefore, it is important to understand the specific contribution of capsule type to fitness during colonization and disease. We speculate that specific serotypes may differ in colonization fitness, which may play a role in serotype distribution in neonatal disease.

## SUPPLEMENTAL MATERIAL

Supplementary Material spectrum.02349-23-s0001.docx Figure S1; Figure S2A, Figure S2B; Figure S3.

ASM does not own the copyrights to Supplemental Material that may be linked to, or accessed through, an article. The authors have granted ASM a non-exclusive, world-wide license to publish the Supplemental Material files. Please contact the corresponding author directly for reuse.
