# Supplementary material for: Capsule production promotes Group B Streptococcus intestinal colonization
Source: Microbiol Spectr. 2023 Sep 21;11(5):e02349-23. doi: 10.1128/spectrum.02349-23 (PMC10655599; doi:10.1128/spectrum.02349-23)

**Supplementary Material.**

**Figure S1. Longitudinal rectal/fecal sampling demonstrates capsule producing A909 outcompetes capsule deficient A909 in a murine model of GI cocolonization.** Preweaning C57BL/6J mice were orally inoculated with a 1:1 mixture of A909 WT and A909Δ*cpsE*. Rectal swab sampling was performed longitudinally in pre-weaning mice (n=8) at predetermined time intervals. Immunoblot with type Ia primary antibody was used to differentiate between capsule A909 WT and A909 Δ*cpsE*. Data points represent geometric mean competition indices and error bars represent 95% confidence intervals

**Figure S2. Longitudinal rectal/fecal sampling demonstrates an advantage of serotype capsule Ia over serotype capsule III in wild type and isogenic mutant backgrounds.**

**A**. Preweaning C57BL/6J mice were orally inoculated with 1:1 mixture of A909 Rev and A909 CS. Rectal swab sampling was performed longitudinally in pre-weaning mice (n=8) at predetermined time intervals.

**B.** Preweaning C57BL/6J mice were orally inoculated with 1:1 mixture of A909 WT and COH1 WT. Immunoblot with type Ia and type III primary antibodies were used to differentiate between capsule Ia and III. Data points represent geometric mean competition indices and error bars represent 95% confidence intervals.

**Figure S3. Immunofluorescent staining for biological and technical controls**

**A, B.** Representative images GBS-specific immunofluorescent antibody staining in colon of sham control and technical control respectively. GBS are visualized using a GBS-specific antibody stain (red) and cell nuclei are stained with DAPI (blue).


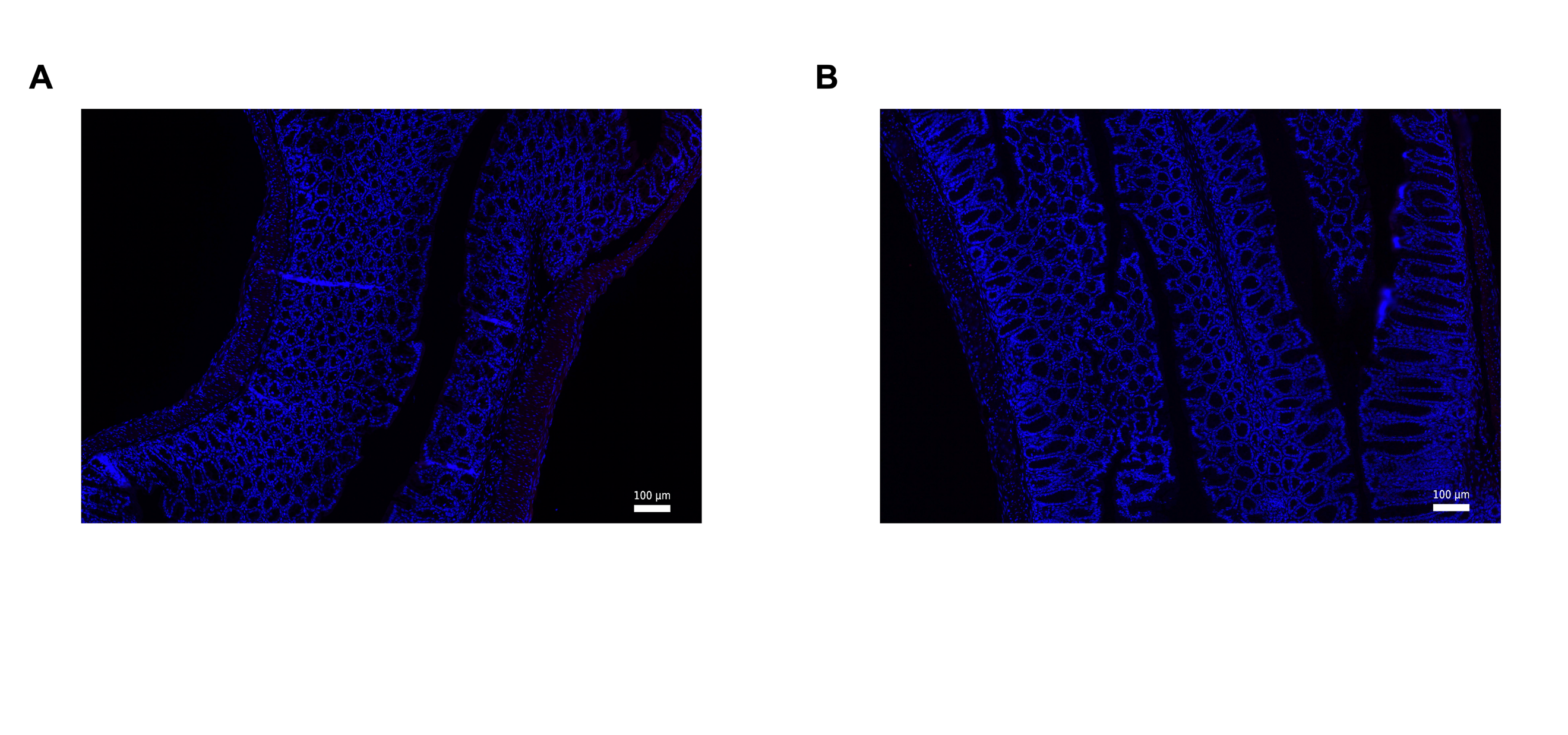

Supplement: Supplementary Material — Figure S1; Figure S2A, Figure S2B; Figure S3. [file spectrum.02349-23-s0001.docx]
